# A Perspective on the Experimental Techniques for Studying Lamins

**DOI:** 10.3390/cells6040033

**Published:** 2017-10-10

**Authors:** Ilaria Pecorari, Daniele Borin, Orfeo Sbaizero

**Affiliations:** Department of Engineering and Architecture, University of Trieste, Via Valerio 10, 34127 Trieste, Italy; ILARIA.PECORARI@phd.units.it (I.P.); dborin@units.it (D.B.)

**Keywords:** lamins, experimental techniques, microscopy, spectroscopy, mechanics

## Abstract

Lamins are type V intermediate filaments that collectively form a meshwork underneath the inner nuclear membrane, called nuclear lamina. Furthermore, they are also present in the nucleoplasm. Lamins are experiencing a growing interest, since a wide range of diseases are induced by mutations in the gene coding for A-type lamins, globally known as laminopathies. Moreover, it has been demonstrated that lamins are involved in other pathological conditions, like cancer. The role of lamins has been studied from several perspectives, exploiting different techniques and procedures. This multidisciplinary approach has contributed to resolving the unique features of lamins and has provided a thorough insight in their role in living organisms. Yet, there are still many unanswered questions, which constantly generate research in the field. The present work is aimed to review some interesting experimental techniques performed so far to study lamins. Scientists can take advantage of this collection for their novel investigations, being aware of the already pursued and consolidated methodologies. Hopefully, advances in these research directions will provide insights to achieve better diagnostic procedures and effective therapeutic options.

## 1. Introduction

In eukaryotic cells, the nuclear envelope (NE) acts as a physical barrier between cytoplasm and nucleoplasm. NE is a bilayered lipid membrane with a highly organized and complex structure, which comprises the inner (INM) and the outer (ONM) nuclear membranes, the nuclear pore complexes (NPCs) and the underlying nuclear lamina. The NPCs provide the sole exchanging channels between cytoplasm and nucleus. Nuclear lamina is a dense protein meshwork underneath the INM and is mostly comprised of type V intermediate filaments known as lamins. In vertebrates, lamins can be classified in two major groups (A- and B-type lamins), depending on their biochemical and sequence characteristics [1]. In mammals, the *LMNA* gene encodes all A-type lamins (lamins A, C, Aδ10, and C2), by alternative splicing. The *LMNB1* gene encodes lamin B1, whereas *LMNB2* encodes lamins B2 and B3. In invertebrates, the scenario is a bit different: in *Drosophila melanogaster*, for instance, there are two genes coding for lamin Dm and lamin C, which can be associated with the B- and A-type lamins of vertebrates, respectively.

Lamin monomers have tripartite structural organization, consisting of a α-helical central rod domain contiguous with a short globular *N*-terminal head and a longer *C*-terminal tail domain [2]. Similar to other intermediate filaments, lamins polymerize to form dimers and other higher ordered structures [3].

Besides their presence in the nuclear lamina, lamins appear throughout the nucleoplasm in the form of foci, showing a different mobility compared to their nuclear lamina counterpart. In particular, nucleoplasmic A-type lamins were demonstrated to be more dynamic than those incorporated in the meshwork [4].

Lamins are involved in several processes, including maintenance of mechanical cell integrity and stability, modulation of gene expression, DNA replication and apoptosis. Due to this fundamental role, alterations in lamins are likely to be associated with pathological phenotypes. For instance, mutations in *LMNA* have been attributed to a class of disorders, collectively referred to as laminopathies; these range from muscular dystrophies to premature ageing disease to cardiomyopathies. Phenotypes and diseases are not mutually exclusive, but can overlap and lead to unique pathological conditions. Furthermore, lamins have been extensively studied in cancer, as thoroughly reviewed in [5]. Despite the already well-established role of A-type lamins in the onset of laminopathies, the involvement of B-type lamins in healthy and pathological conditions is still in its scientific infancy; a detailed state-of-the-art is provided in [6].

**Figure 1:**
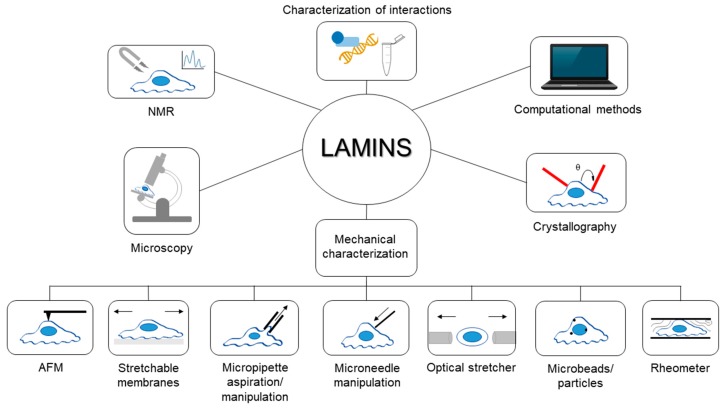
General overview of the techniques described in the review. In the figure, samples have been identified as cells, even if some techniques, like rheological ones, are performed also with lamin solutions or different specimens.

Since their discovery, lamins have been studied exploiting various experimental techniques and procedures from different perspectives; for instance, in recent years, the biomechanical behavior of lamins and their involvement in mechanotransduction has raised great interest in the scientific community. Covering the extremely wide literature in the field is outside the main goal of the present work. Rather, this review aims to collect some interesting examples and highlight the applications of each technique for studying lamins and their role in physiological and pathological conditions. A general overview of the techniques described in this paper is shown in Figure 1. Hopefully, this collection of methodologies will provide readers with a broad foundation of the investigations surrounding lamin and how they can be combined to better understand its properties and role in disease.

## 2. Nuclear Magnetic Resonance (NMR) Spectroscopy

Nuclear magnetic resonance spectroscopy is commonly used to determine the structure of organic compounds. It relies on the phenomenon by which certain atomic nuclei absorb and re-emit electromagnetic radiation when they are subjected to a magnetic field. This method has been applied to investigate lamins. For instance, the globular structure of the carboxyl-terminus of human lamins A/C was resolved via nuclear magnetic resonance spectroscopy in 2002 [7], determining the immunoglobulin fold of type “s” of the C-terminus. Moreover, NMR technique allowed studying the structure and thermal stability of three mutants related to laminopathies.

In the work published by Stierlé et al. [8], a peptide encompassing amino acids 411–533 of human lamins A and C (on the *C*-terminal end of the proteins) was investigated by NMR spectroscopy. The in vitro binding between DNA and this peptide was analyzed, and regions involved in the interaction were identified.

NMR has been further applied to study lamins: the tail domain of lamin C was incubated in 293T cell extracts and NMR spectra were collected to follow modifications and binding events of the protein [9].

## 3. Microscopy

Generally, microscopy techniques can be mainly divided into three categories: optical, electron, and scanning probe microscopies. In optical and electron microscopy, an incident electromagnetic radiation interacts with the sample under investigation; the resulting scattered radiation is collected to reproduce the image of the specimen at higher magnification than the one observed with naked eye. On the other hand, scanning probe microscopy exploits the interactions between a probe and the surface of the sample in order to image sub-micrometer objects. Atomic force microscopy (AFM) is one of the most common scanning probe techniques and is described in detail in Section 5.1.

Among all the optical microscopies, fluorescence microscopy is extensively used in the study of biological samples. This technique takes advantage of the capability of some compounds (known as fluorophores) to re-emit light at longer wavelengths, upon light excitation. The fluorescence phenomenon is utilized to create the image of the specimen.

In the following sections, some applications of different microscopy techniques to study lamins will be discussed.

### 3.1. Electron Microscopy

In contrast to optical microscopy and visible light, electron microscopy (EM) takes advantage of an accelerated beam of electrons to illuminate the specimen. A comprehensive overview of the first studies performed with electron microscopy on lamins is provided in the introductive part of [10]. Although, it is worthy to mention the work of Fawcett [11], which paved the way for the studies on lamins. The author identified a layer in the nuclei of cells derived from several vertebrates (namely, ventral nerve cord of the leech, the germinal epithelium and interstitial tissue of the cat testis, the guinea pig ductus epididymidis, and the intestinal mucosa of the Congo eel), that was associated with the “fibrous lamina” previously observed in various invertebrates. Electron microscopy contributed to establish the “intermediate filaments nature” of lamins as per the observation of isolated NEs of *Xenopus* oocytes and purified lamins A, C and B from NEs of rat liver [12]. Furthermore, in 2008, Cohen et al. [13] wrote a book chapter on the study of nuclear lamina in a specific invertebrate model organism, the *Caenorhabditis elegans*.

When samples are labelled with antibodies against a required antigen and gold particles, they can be analyzed via the so-called immunoelectron microscopy. This technique was used to detect lamin A/C in quick-freeze freeze-substituted mouse anterior pituitary cells [10]. Quick-freeze freeze-substitution is an electron microscopy preparation procedure, which works better than conventional, chemical fixation protocols for the localization of both nuclear lamina and its major constituents. Immunoelectron microscopy was also exploited to study how lamins are organized in both normal (NH) and transformed (PHN) rat hepatocytes [14]. The authors showed the regularity in the distribution of lamin epitopes in normal cells, while two-dimensional local order was in the transformed counterpart.

Conventional EM and immuno-EM were combined and applied to study cardiac tissue samples from dilated cardiomyopathy (DCM) patients with atrioventricular block (AVB) caused by mutations in *LMNA*. Electron microscopy allowed Verga and coworkers [15] to discover that nuclei of cardiomyocytes (CMs) from patients carrying *LMNA* mutations display an irregular shape. Researchers explored different procedures to prepare samples for immunoelectron microscopy, finally demonstrating that CMs, interstitial and vascular cells of control specimens (both DCM patients not ascribed to *LMNA* mutations and normal donors) are intensely stained, whereas samples with mutant A-type lamins retained the immunolabeling only in interstitial and vascular cell nuclei (but still, less than control counterpart).

In addition, electron microscopy can be used to produce 3D images of a sample, as it is held at cryogenic temperatures and tilted in space during 2D image acquisition. 2D images can be combined through computational methods to obtain 3D reconstructions of the specimen, similarly to CT scans. This technique is commonly known as cryo-electron tomography, and it has been used for characterizing the lamin meshwork of vimentin-null mouse embryonic fibroblasts (MEFs) at molecular resolution [16]. In particular, lamin filaments appeared as globular-decorated fibers, and A- and B-type lamins were shown to assemble into tetrameric filaments.

Often, researchers combine electron microscopy investigations with other techniques, e.g., atomic force and fluorescence microscopy, to better understand the role of lamins. Kaufmann et al. [17] used transmission electron microscopy to analyze thickness and morphology of the lamina in isolated oocyte nuclei from *Xenopus laevis*. Comparison was made among nuclei injected with mutant (E145K) human lamin A, wild-type lamin A or not injected at all. Although lamina in non-injected nuclei was hardly detectable, the layer in nuclei expressing exogenous lamin showed different characteristics, i.e., the presence of paracrystal formations. Immunoelectron microscopy supported the discussion of the results obtained by Moir et al. in 2000, who observed nucleoplasmic lamin staining in PAM (embryonic mouse epidermal cells) and BHK-21 (hamster kidney fibroblasts) cell lines, both expressing green fluorescent protein (GFP)–lamin fusion proteins; nonetheless, data were mentioned, but not shown [18]. Recently, EM-based techniques allowed Zhironkina et al. to resolve the continuous layers formed by lamin A and B1 and the various concentrations of lamins in different areas [19]. Noteworthy, authors also used the correlative light-electron microscopy, which results from the combination of an optical microscope with an electron microscope, to observe the contact regions between chromatin and nuclear envelopes at several time points. Finally, a combination of microscopies, including EM, was exploited to analyze the effects of lamin B1 depletion in human cervical carcinoma (HeLa) cells [20]. For electron microscopy analyses, cells were subjected to either actinomycin D or DRB (5,6-dichloro-1-β-d-ribofuranosylbenzimidazole) treatments, which mimicked the characteristics acquired with lamin B1 depletion. In controls, active centres in the nucleoli showed their classical structure, which was altered due to the two aforementioned treatments, especially with actinomycin D.

### 3.2. Fluorescence Recovery after Photobleaching (FRAP)

Fluorescence recovery after photobleaching is a common technique used to characterize the mobility of molecules within tissues or cells through their diffusion kinetics. A region of the investigated sample is subjected to high intensity illumination, so the fluorescence lifetime of the fluorophores in that area rapidly elapses. Afterwards, the non-bleached compounds diffuse in the specimen, replacing the bleached fluorophores. The mobility of the fluorescent molecules can be then inferred upon the diffusion process. A comprehensive description of the protocols to assess the mobility of lamins via FRAP is provided in [21].

Over the years, this method has been extensively applied to study lamins. Bleaching techniques, namely FRAP and fluorescence loss in photobleaching (FLIP), confirmed the immobility of A-type lamin-GFP proteins in both the nuclear lamina and tubules of CHO-K1 (Chinese hamster ovary) cells [22]. Notably, the lamin-GFP found in the nucleoplasm was already thought as “a pool of soluble intranuclear A-type lamins next to the immobile fraction”. In 2004, the group of Gilchrist [23] studied the dynamics of lamin A within the nuclear lamina and the nucleoplasm. HT1080 cell line (human epithelial cells from fibrosarcoma) expressing GFP tagged wild type and mutant lamin A were analyzed via FRAP. Four mutations were taken into account, namely R482W, L85R, L530P and N195K, in order to determine if different diseases can be correlated with different dynamic behaviors. Indeed, R482W (familial partial lipodystrophy, FPLD) mutant lamin was shown to behave like the wild-type counterpart; an increased mobility within nuclear lamina was observed in L85R (DCM) and L530P (autosomal dominant Emery-Dreifuss muscular dystrophy) mutants. N195K (DCM) dramatically raised the dynamics of lamin A in both the nucleoplasm and the nuclear lamina. Data for wild-type, L530P and N195K were also confirmed by FLIP. The mobility of certain mutant proteins was studied in HEK 293 (human embryonic kidney) cells by Piekarowicz and colleagues; although, mutants exhibited more or less the same low mobility [24]. It is worthy to highlight that the mutant lamin A Δ50, which is permanently farnesylated and probably immobilized to the nuclear envelope, showed the lowest mobility (about 10% lower than that of lamin A). However, no statistical difference was found.

Other mutations in *Drosophila* lamins were investigated by Zaremba-Czogalla et al. [25] to achieve a better comprehension of the role played by several phosphorylation sites, since these are strictly correlated with lamin functions. Via FRAP, authors revealed the higher mobility of lamin Dm T^435^E compared to other mutants or wild-type lamins. Additionally, phosphorylation sites have been studied in HeLa cells expressing GFP-lamin A using the wild type protein or phosphomimetic and phosphorylation-deficient mutants [26]. Indeed, differences in the mobility of lamin constructs were identified depending on the mutations, thus strengthening the relevance of phosphorylation sites. Moreover, it was demonstrated that some phosphomutant lamin A constructs and their wild type counterpart have different mobility, regardless of the stiffness of the substrate to which cells adhere [27]. It is interesting to underline that wild type GFP-lamin A was more mobile in mesenchymal stem cells seeded on soft substrates (0.3 kPa) than on stiffer ones (40 kPa) [27]. The phosphorylation issue has been explored even in *Xenopus laevis*; as an amphibian, this animal model has the so-called lamin B3, which is similar to B-type lamins, but has a sequence that can be referred to A-type lamins [28]. In the recent work of Edens et al., a new phosphorylation site for conventional protein kinase C (cPKC) was discovered. FRAP analyses revealed that phosphorylation at this site leads to an increase of the lamin dynamics [29].

Besides mutations, the depletion of lamins was studied in order to assess to what extent lamins contribute to the stability of the lamina network. FRAP analysis was performed in HeLa cells co-transfected with RNAi vectors and vector expressing GFP-lamin A/C or B. A change in the recovery halftime, caused by the depletion of either A- or B-type lamins, was discovered [30]. This finding confirmed again the role played by both lamin classes to the stability of nuclear lamina.

Lastly, the bleaching technique revealed the enhanced mobility of eGFP-tagged lamin A in murine NIH3T3 fibroblasts after perturbation of F-actin organization, triggered by either the patterning of the substrate or the polymerization state of actin itself [31].

### 3.3. Fluorescence Correlation Spectroscopy (FCS)

FRAP analyses are often associated with another technique, namely fluorescence correlation spectroscopy. FCS detects the little, spontaneous fluctuations in the fluorescence intensity of fluorescent particles and it has been included in this section since, like FRAP, it takes advantage of optical microscopy and the fluorescence phenomenon. In the already reported work of Takeshi et al. [21], protocols and procedures for studying lamins via FCS are thoroughly described.

Fluorescence correlation spectroscopy contributed to confirm that A- and B-type lamins build different, but interconnected, structures within a cell. Indeed, more than 90% of nucleoplasmic GFP-lamin A and GFP-lamin C exhibited a rapid mobility, whereas GFP-lamin B1 and GFP-lamin B2 were somewhat static [32]. Although silencing of either lamin B1 or lamin B2 led to an increase of the mobility of the so-called “slow fraction” of nucleoplasmic GFP-lamin A, no variations emerged for fast fraction of GFP-lamin A, and GFP-lamin C.

The aforementioned paper of Zaremba-Czogalla cited, but did not show, FCS experiments to support their findings about the increased mobility of lamin Dm mutant T^435^E [25]. Similarly, Kochin and coworkers combined the results derived from FRAP and FCS analyses; the latter assessed the rapid nucleoplasmic mobility of wild-type lamin A and its mutants [26]. Nevertheless, FCS highlighted some significant differences in the diffusion coefficients of the above proteins. FCS has been coupled with FRAP in the study of Toh et al., in which FCS revealed different dynamics of lamin A due to the cell geometry (induced by physical constraints) and the perturbation of actin (caused by drug administration) [31].

### 3.4. Further Hints about Microscopy

In this microscopy section, it is relevant to discuss the paper published by Shimi et al. in 2015, where the supramolecular structure of lamin A, C, B1, and B2 in mouse embryo fibroblast nuclei was presented by means of superresolution microscopy, a technique that can overcome the Abbe diffraction limit [33]. Via computational image analysis, authors discovered that each lamin isoform builds a separate meshwork within the nuclear lamina, even if some similarities between them have been observed. To further investigate the interactions between these structures, comparison among data on MEFs derived from wild type, *Lmna*, *Lmnb1*, and *Lmnb2* knockout mice was performed, highlighting the alteration of remaining networks when lamin A/C or B1 expression is null. No change in the meshwork formed by other isoforms was identified when lamin B2 is absent. Data of Shimi and coauthors have been recently confirmed: A- and B-type lamins have been found to assemble in separate filaments networks in immortalized *Lmna* ^−^/^−^ mouse adult fibroblasts (MAFs) [34]. Furthermore, the superresolution microscopy allowed resolving the attachment sites for nuclear pore complexes within the nuclear lamina.

Another kind of microscopy deserves to be mentioned for studying lamins, namely the differential interference contrast (DIC) microscopy. This technique exploits interferometry to enhance the contrast of unstained, transparent specimens. Combining DIC with particle tracking methods, several lamin-rich and -poor regions were identified within human lamin B1 gels, because the first remain focused while the others do not [35].

## 4. Crystallography Techniques

Over the years, crystallography techniques allowed the scientific community to determine the structure of a broad range of molecules, such as proteins. By interacting with the sample of interest, an incident beam is diffracted, and the features of the diffracted beams provide structural data of the specimen. Applying this technique, some structural details of lamins have been revealed. The 3D crystal structure of the *C*-terminal tail domain of lamin A/C was described at 1.4 Å resolution by the group of Dhe-Paganon, together with a speculation about mutations leading to muscular dystrophy or lipodystrophy, which are in the protein core or in a corner of the domain, respectively [36]. Data from this study were used as an initial model for assessing the crystal structure of carboxyl-terminal domain of R482W mutant human lamin A/C at 1.5 Å resolution [37]. This mutation has been already mentioned throughout the present work, since it causes FPLD, and the paper of Magracheva et al. [37] is just one among the numerous others that tried to shed light on mechanisms behind laminopathies.

This methodology was also exploited to determine the structure of the lamin rod. Indeed, most of the *C*-terminal coiled-coil segment 2B (one of the constitutive parts of the central rod domain) was observed at 2.2 Å resolution [38].

Other studies and details about crystallographic studies of lamins can be found in [39].

## 5. Mechanical Characterization Techniques

As outlined in the introduction, lamins are widely accepted to play a fundamental role in the structural properties of the nucleus and in mechanotransduction, namely the conversion of mechanical stimuli in biochemical signals. This achievement was accomplished after the introduction and development of several techniques, which will be hereby briefly presented. Lastly, their application to the study of lamins will be highlighted.

### 5.1. Atomic Force Microscopy

In atomic force microscopy, the interactions between a tip mounted on a cantilevered spring and the sample are transduced into an electrical signal. The cantilever beam is mounted on a X,Y,Z piezo scanner to investigate different portions of the sample (X and Y directions) while controlling the applied force or distance of the probe with a feedback loop acting on Z direction. The acquired signal is converted into a topographical image of the specimen, and/or is associated with the mechanical properties of the investigated object. This technique senses force in the order of pN, with a sub-nanometer spatial resolution. The numerous potentialities of AFM for biological investigations have been recently reviewed by our group [40].

AFM can be used as an imaging tool for morphological characterization or to investigate the mechanical properties of single molecules, subcellular portions (e.g., the nucleus) or whole cells.

Recently, our group discussed the assessment of mechanical behavior of lamins via AFM [41], so in this current work we will consider other studies that demonstrate additional ways that AFM can be applied to study lamin.

Foeger et al. [42] exploited the AFM as an imaging tool to study the in vitro assembly of lamin B from *Caenorhabditis elegans*, focusing on the formation of intermediate assemblies.

Bera and colleagues [43] used AFM for single molecule force spectroscopy to elucidate the role of domain 1B and 2B in the rod portion of lamin A. The 1B domain was identified as the main contributor for the lamin A network elasticity. Interestingly, authors combined other techniques (scanning electron microscopy, differential scanning calorimetry, rheology and in silico methods) to demonstrate that 1B domain forms networks, whereas the 2B domain assembles into short structures.

The E145K mutant form of lamin A, which is associated with the Hutchinson-Gilford progeria syndrome (HGPS) was shown to increase the nuclear stiffness not only in *Xenopus* oocytes [17], but also in dermal fibroblasts from a 4-year old HGPS patient, when compared to those from a 10-year old healthy donor [44]. In both cases, TEM imaging did not display significant differences in NE thickness between mutants and controls. Similarly, the autosomal dominant leukodystrophy (ADLD), characterized by an over-expression of lamin B1, resulted in the nuclear stiffening of human skin fibroblasts [45]. Notably, in this study the authors used also patch-clamp experiments on isolated nuclei, showing a reduction in the probability of open nuclear ion channels in ADLD patients.

Haase and colleagues [46] combined AFM and laser scanning confocal microscopy to study the intrinsic, anisotropic nuclear deformation of several cell lines when squeezed by the AFM probe. In intact cells, the nuclear deformation is impeded by cytoskeletal structures, but reduction of lamin A/C levels and altered chromatin organization were identified as responsible for nuclear isotropic deformation.

Recently, AFM analysis on nuclei of bone marrow stem cells from adult rats demonstrated the role of osteopontin (OPN) in reducing the expression level of lamin A/C and lowering the nuclear stiffness [47].

Isolating nuclei from whole cells to study can potentially introduce artefacts during the extraction process, so other studies have characterized the influence of lamin by instead probing the nuclear portion of whole cells. In the work of Nagayama et al. [48], atomic force and confocal microscopy were used to compare the nuclear stiffness of porcine aortic smooth muscle cells (SMCs) and HeLa cells. The lamin A/C layer of NE was thicker in SMCs, resulting in stiffer nuclei compared to that of HeLa cells. In this study, cells were pre-treated with cytochalasin D to exclude the influence of F-actin on the stiffness, obtaining values comparable to those on isolated nuclei (and half than those of intact cells).

The effect of differentiation of bovine and human mesenchymal stem cells (MSCs) on nuclear mechanical properties was investigated by Heo and colleagues [49], resulting in a stiffening of nuclei caused by lamin A/C restructuring and increased heterochromatin production. Interestingly, in this case the actin depolymerization gave the same stiffness as intact cells.

### 5.2. Mechanical Strains through Stretchable Membranes

This technique exploits deformable membranes (usually made of silicone with a proper coating, e.g., fibronectin) that are stretched uni- or bi-axially to evaluate the pliability of cells cultured on them. Nuclear stiffness is inferred from the nucleus strain (usually measured by fluorescence) induced by static deformation, whereas cyclic deformation is used to assess cell viability.

In 2004, Lammerding and colleagues [50] used both static and cyclic deformations to demonstrate that lamin A/C-deficient MEFs have more deformable nuclei, reduced mechanotransduction and impaired viability compared to wild type cells. The same approach was used to assess the specific role of different lamin subtypes on nuclear mechanical properties of MEFs: lamin A/C deficiency induced abnormal nuclear shape, reduced stiffness and affected cell viability under strain. Lamin A deficient MEFs displayed less misshapen nuclei, lower stiffness reduction and unchanged viability, whereas cells missing lamin B1 showed unaltered nuclear mechanics but more frequent nuclear blebs, thus outlining the different functions of lamin A/C (nuclear stiffness) and lamin B1 (integrity) [51]. Skin fibroblasts from HGPS patients showed increased nuclear rigidity with culture time (restored to control values by farnesyltransferase inhibitors), decreased viability and impaired mechanotransduction [52]. Moreover, Zwerger and colleagues tested both skin fibroblasts from laminopathic patients and *Lmna* ^+^/^−^ MEFs expressing different lamin mutations, showing that those exhibiting muscular phenotypes (as dystrophy and DCM) have more deformable nuclei, due to an impairment of the nuclear lamina assembly [53].

In the already cited work of Heo et al. [49], aligned poly(ε-caprolactone) nano-fibrous scaffolds were used as deformable membranes for MSCs, showing that differentiation is enhanced by dynamic loading conditions and it lowers nuclear deformability.

### 5.3. Micropipette Aspiration/Manipulation

Micropipette aspiration is widely used to characterize the viscoelastic properties of investigated samples, which can be either intact cells or isolated nuclei. Specimens are fluorescently labelled and a glass microcapillary, with an internal diameter at least 2 to 3 times lower than the dimension of the probed element, applies a negative gradient pressure to aspirate the sample. Using fluorescence microscopy, the deformation of the cell/nucleus inside the micropipette is accurately measured as function of time and applied pressure. The creep compliance, namely the viscous and elastic properties of the sample, can be then calculated by theoretical models.

Micropipette aspiration was applied by Dahl and colleagues [54] on isolated nuclei of TC7 cells to investigate the role of both chromatin and lamin B1 in the nuclear viscoelastic properties. Interestingly, authors used a swelling nuclear assay to separate the chromatin and lamin contributions. In both swollen and unswollen conditions, creep compliance assessment demonstrated a power law dependence of nuclear deformability with time (confirmed also by AFM on unswollen nuclei). Authors demonstrated that the nucleus has a stiff response for short-time stresses, whereas it becomes more deformable in prolonged stress conditions. The same approach was applied on dermal fibroblasts from HGPS patients, showing that in swollen nuclei from HGPS cells, lamin A was less prone to deformation than in that of healthy controls, forming wrinkled structures outside the pipette and not being able to recover the nuclear original size after unswelling [55]. In this study, polarization microscopy displayed that WT lamin A is homogenously distributed, while HGPS lamin forms locally aligned micro-domains, thus potentially explaining its altered response to mechanical stress.

Micropipette aspiration was used on intact HeLa cells and their isolated nuclei to study the mechanical response of the NE to increasing applied pressures [56]. The lamina layer was modeled as an elastic, two-dimensional solid material, whereas the nuclear membranes exhibited a fluid-like behavior.

Moreover, the effect of differentiation on deformability of human stem cells, both embryonic and hematopoietic, was probed with this technique [57]. The nuclear compliance was strongly affected by lamin A/C content, as confirmed by human epithelial A549 cell line with induced lamin deficiency, whereas the rheological properties of the nucleus were mainly ascribed to nucleoplasm and chromatin.

A further characterization of the role of lamin subtypes was proposed by Swift and colleagues [58], probing several cell lines with different expression levels of lamin A. In this work, the *LMNA* expression resulted to increase with the substrate stiffness. Authors confirmed a reduced creep compliance with high *LMNA* content, and verified a linear dependence of both elastic and viscous moduli with the lamin A:B ratio. The viscoelastic response was modeled considering B-type lamins as the elastic element (spring), in series with the viscous lamin A (dashpot). The ratio of viscous and elastic response defines the characteristic elongation time, with a power-law dependence on the lamin A:B ratio. The role of lamin B1 as a spring was also confirmed in the work of Shin et al. [59], where its 50% knockdown in U251 cells increased the elongation time 30-fold compared to wild-type nuclei. The effect of the lamin A:B ratio was further investigated by Harada and colleagues on cancerous and mesenchymal stem cell lines [60]. Low A:B ratios favor nuclear deformation and, consequently, cell migration, but also make cells more exposed to damages from shear stresses. Furthermore, micropipette aspiration was used to compare the deformability of two melanoma cell lines (WM35 and Lu1205), which was strongly reduced by inducing the expression of Δ50 lamin A, associated with HGPS [61].

In 2015, Booth and colleagues demonstrated with this technique that progerin (a mutant form of lamin A, related to HGPS) expression in different cell lines induced nuclear stiffening [62]. Lastly, the force necessary to deform the nuclei of several adherent cell lines was quantified by micropipette aspiration in the work of Neelam et al. [63], in which the role of lamin A/C in nuclear shape conservation was outlined, as well as the impaired connection between nucleus and cytoskeleton in *Lmna* ^−^/^−^ MEFs.

In micropipette manipulation, a single cell or isolated nucleus is captured between two micropipettes, one acting as puller and the other one is calibrated to convert the micropipette deflection into the force required to deform the specimen. Capturing requires low aspiration pressures, thus avoiding the strong non-linear and non-homogeneous strains of micropipette aspiration. An application of this technique on isolated nuclei of different cell lines is provided in the work of Stephens et al. [64]. Chromatin was demonstrated to control the morphology and the strain response in the low regime deformation (<3 μm) of the nucleus, whereas lamin A/C plays a key role in the mechanical behavior at higher deformations.

### 5.4. Microneedle Manipulation

This technique is used to assess the mechanical transmission of forces between cell compartments, such as nucleus and cytoskeleton. A micrometric capillary is inserted into the cytoplasm of intact cells, close to the nucleus, and pulled toward the periphery. By fluorescence imaging, the relative displacements of cell compartments are measured. An example of this approach is reported in the already cited work of Zwerger et al. [53], demonstrating that the studied lamin mutations impaired the mechanotransduction machinery, reducing the force transmission between nucleus and cytoskeleton.

### 5.5. Optical Stretcher

Here, a low-power laser source is employed to create a dual beam optical trap, thus immobilizing cells in suspension. The increase of laser power induces a cell stretching, revealing the time-dependent mechanical response of the cell. This tool has been recently used by Kolb et al. [65] to probe the nuclear properties of both adult mouse fibroblasts (adherent cell line) and K562 cells (suspension cell line) with different nuclear lamin A levels. Authors demonstrated that, in physiological conditions, the nucleus deforms less than the surrounding cytoskeleton. A reduction in the *LMNA* content increases mainly the nuclear deformability compared to that of the whole cell.

### 5.6. Microbeads/Microparticles

Several techniques exploit the use of micro- or nanoparticles to probe locally the mechanical properties (mainly the viscoelastic behavior) of the cells.

Particle tracking micro-rheology monitors the Brownian motion of sub-micrometer fluorescent beads injected into cytoplasm or nucleoplasm. Similarly, in ballistic nano-rheology, cells are bombarded by fluorescent nanoparticles (around 100 nm in diameter), which penetrate the cellular membrane by impact and not endocytosis. In both techniques, the time-dependent mean squared displacement is calculated using fluorescence image analysis, by which the local viscoelastic properties are derived, namely elastic and viscous moduli, shear viscosity and creep compliance.

Particle tracking micro-rheology was exploited by Panorchan and colleagues [35] using functionalized microbeads on lamin B1 networks. In combination with differential interference contrast microscopy, authors observed the formation of extremely porous networks with areas of high and low density of lamin B1, thus confirming the structural role of this lamin subtype in the NE. This technique was also used in the previously cited work of Booth et al. [62], where intranuclear particle tracking in cells expressing progerin showed a more compliant nuclear interior. Such softening, and the demonstrated nucleoskeletal stiffening, was suggested to be responsible for the altered mechano-responsiveness of HGPS tissue.

Ballistic nano-rheology was applied to *Lmna* ^−^/^−^ MEFs, showing that lamin deficiency can alter cytoplasm properties, reducing both elasticity and viscosity, similar to drug induced F-actin and microtubule depolymerization [66].

Magnetic microbeads, usually with a proper coating (e.g., fibronectin) to adhere to the cell surface, are used to probe viscoelastic cell properties, monitoring their displacement as function of an applied magnetic field (magnetic tweezers or twisting magnetometry techniques). In [50], a sinusoidal magnetic field was applied to paramagnetic beads on *Lmna* ^−^/^−^ MEFs, resulting in a reduced cytoskeletal stiffness induced by lamin deficiency. The same approach was applied to skin fibroblasts from HGPS patients but did not show differences in cytoskeletal properties compared to controls [52].

### 5.7. Rheology

The mechanical properties of lamin networks can be characterized on a “macroscopic” scale using rheometers. Lamin solutions are loaded between two plates of different geometries (namely, parallel plates or cone-and-plate geometries) and induced to polymerize. The application of shear stresses (e.g., sinusoidal with frequency sweep) and the acquisition of the mechanical response enables the assessment of the viscoelastic properties of the sample. As an example, the properties of lamin B1 networks and their role in the NE disassembly during mitosis were studied with this method [67]. Authors found that such networks have a predominant solid-like behavior, with strain hardening and a very high shear resilience, which exceeds the force exerted by microtubule spindle at the beginning of mitotic division. Thus, the action of a biochemical process, which induces lamin B1 network weakening, was deemed necessary to achieve the NE breakdown during mitosis. The same approach applied to the lamin A network showed a strain-softening effect, while mutant lamins associated with DCM displayed a reduction in both the elastic and viscous moduli compared to the wild-type form [68].

Isolated nuclei of A549 cells with different expression levels of *LMNA* were probed on a cone-and-plate rheometer, showing conformational changes of lamin structure caused by shear stress. Moreover, increasing levels of *LMNA* can prevent chromatin damages within the nucleus [58].

### 5.8. Other “Mechanical” Techniques

The role of lamins on nuclear and cellular mechanics has also been characterized through several indirect mechanical approaches, which will be reported briefly here.

#### 5.8.1. Cell Migration and Wound Healing Assays

The effects of lamin deficiency and/or mutation on the cell migration capability have been tested both in 2D and 3D assays. In the first case, the wound healing assay is commonly applied by scratching a cell monolayer with a glass micropipette and counting the cells that migrate into the “wound” over time. This assay has been used to demonstrate the reduced migration in *Lmna* ^−^/^−^ MEFs [66] or in cell lines with high lamin A:B ratios [60]. The increased migration speed of HGPS skin fibroblasts after farnesyltransferase inhibition was also shown [52]. In this study, in order to distinguish the proliferation and migration effects at the wound edge, cell migration was assessed by tracking the movement of cells plated at low density.

3D cell migration assays usually exploit commercially available inserts with micropore filters, which are coated with extracellular matrix components and/or cellular monolayers to mimic physiological barriers to migration. Cells are seeded on one side of the filter, whereas on the other side a chemoattractant compound induces cell migration. After a fixed time, cells that have passed through the membrane are counted. This approach was used to study the effects on migration induced by osteopontin in MSCs [47] and by the lamin A:B ratio of different cell lines [60]. The migration tendency of melanoma cells was reduced after inducing the expression of Δ50 lamin [61]. In the last study, a flow chamber was used to circulate tumor cells, while the microporous membrane was coated with a layer of endothelial cells.

Another cell migration assay was proposed as an alternative to the commercial micropore systems. A 3D microfluidic device with variable channel dimensions and adhesive coatings was designed to correlate cell migration with nuclear dimension, stiffness, cell adhesiveness and contractility of four different cancer cell lines [69]. In the work of Davidson et al. [70], two different 3D microfluidic devices were used to study cell movement through micron-sized asperities, both for passive perfusion and active migration induced by chemoattractant gradient.

#### 5.8.2. Patterned Substrates

Micropatterned substrates were exploited to study the relationship between nuclear deformability (assessed by means of optical and/or fluorescence microscopy) and lamin expression level. Microcontact printing was used by Talwar and colleagues [71] to create fibronectin-coated islands on plastic dishes: lamin A/C deficient nuclei of mouse embryonic stem cells adapted to the underlying triangular geometry, while randomly shaped nuclei increased in number with lamin content.

Micropillar arrays made of polydimethylsiloxane (PDMS) were produced to study the nuclear deformability and the cell proliferation of healthy SMCs and cancerous HeLa cells [48]. In both cell lines, nuclei were deformed between pillars, but the higher content of lamin A/C in SMCs dramatically reduced cell proliferation, thus supporting the speculation that tumor spreading is enhanced by cell deformability.

Recently, Werner and colleagues [72] created a substrate with concave and convex spherical features to study the migratory and differentiation properties of human MSCs. Authors found that concave cavities facilitate cell migration, while lamin A expression level increased passing from concave, to flat, to convex surfaces, thus promoting osteogenic differentiation.

#### 5.8.3. Fluidic Assays

A microfluidic system made of micro-constrictions arrays was recently proposed as a high throughput device to estimate the cell elasticity and fluidity. This system was applied to cells overexpressing lamin A [73] and several cancer cell lines [69].

Flow chamber assay on macroscopic scale was used to evaluate the effect of fluid-induced shear stress on mechanotransduction of lamin A/C knockdown in bovine aortic endothelial cells [74]. In addition, micro-slide flow chambers proved the impaired mechanotransduction in HGPS cells [62].

## 6. Computational Methods

Computational methods are the computer simulations that can analyze the behavior of complex structures via mathematical models. Computation analyses have been applied to study lamins, as stated in the introductive section of the work of Funkhouser et al. [75]. Moreover, the authors developed a mechanical model of the nuclear lamina for investigating the mechanisms that lead to the formation of nuclear blebs, which are generally observed in pathologies associated with misshapen nuclei (e.g., progeria syndrome). The segregation of certain isoforms has been studied on this lamina model, and results have been satisfactorily compared with data from pathological cells (HeLa cells, breast cancer cell line MDAMB231, and fibroblasts derived from a patient carrying the E145K lamin A mutation). Finally, the use of the aforementioned model allowed the authors to speculate that the reduction of the enlargement in the lamina meshwork could prevent bleb formation.

Large-scale molecular dynamics (MD) studies were applied by Zhang and colleagues [76] to simulate the effects of a point mutation (p.Glu358Lys, involved in muscle dystrophy) in the 2B portion of the rod domain of lamin A. No significant alterations of the tensile behavior at the dimer level were shown, suggesting that the considered mutation produces its mechanical effects on higher hierarchical scales.

In the work of Qin et al. [77] the first molecular structure of the lamin A tail domain was simulated. Replica exchange MD simulations were applied to study the effects of Δ50 *LMNA* mutation. The altered structure and the higher stability of the mutant form, confirmed by in vitro experiments, could explain the modification of protein-protein and DNA-protein interaction in the HGPS disease.

Other computational methods have been recently used to identify the *LMNA* mutations that can alter the canonical splice signals and could therefore be pathogenic [78].

## 7. Characterization of the Interactions Established by Lamins

Lamins form complex structures within a cell and interact with many other molecules. Several techniques have been exploited to study the interactions of lamins both with other proteins and chromatin. For instance, the BioID technique was developed to study protein-protein interactions. In BioID, the protein to be studied is fused with a promiscuous *Escherichia coli* biotin protein. The proteins interacting with or near-neighbors of the protein of interest are subjected to proximity-dependent biotinylation, and can then be selectively isolated by affinity capture and identified via mass spectrometry. A detailed description of the protocols to apply BioID technique, especially to the study of lamins, is provided in [79,80]. The technique was first described and validated on human lamin A by Roux et al. [81]. Authors established HEK293 (human embryonic kidney) cells, which can be induced to stably express a “fusion-lamin A”. Endogenous, vicinal proteins underwent biotinylation and were afterwards identified. Among them, proteins like lamina-associated polypeptide 1 and 2 (LAP1 and LAP2), already known to interact with lamin A, were recognized. Additionally, FAM169A was found to be linked to lamin A, and thus was described as a novel constituent of the nuclear envelope. Other applications of BioID for studying lamins have been already reviewed in [82].

BioID allowed the discovery of other components of the NE, such as vaccinia-related kinase 2A (VRK2A). Indeed, BioID ligase was fused with some proteins, including lamin A, and stably expressed in BJ cells (diploid human foreskin fibroblasts) [83]. Therefore, VRK2A was defined as a novel nuclear envelope resident protein, retained at the NE by A-type lamins. In the aforementioned study of Xie et al., researchers observed that Tpr, a NPCs protein, is selectively associated with lamin C by means of BioID [34]. In conclusion, the BioID technique, while still in its scientific infancy, has shown to be a very promising method of investigation, particularly for lamins.

The interactions of lamins with chromatin were mainly detected using DamID (DNA adenine methyltransferase identification) and ChIP (chromatin immunoprecipitation). DamID is extensively applied to localize the binding sites between chromatin and proteins. The procedure uses a fusion protein derived from the protein of interest and methylation of the DNA. Binding between the investigated protein and DNA results in the localization of the methyltransferase at the binding site. Usually, DamID is combined with and/or validated by ChIP, which is also exploited for studying the interactions between proteins and DNA. DNA and protein are usually crosslinked, thus these complexes need to be sheared and then immunoprecipitated with protein-specific antibody. The sequence of the DNA fragments can then be identified, showing the regions in the genome which are bound to the protein of interest.

Among these two techniques, DamID was the first to identify and characterize the lamina-associated domains (LADs) [84], which were then confirmed by ChIP [85,86]. Besides LADs, ChIP sequencing revealed the existence of distinct lamin-interacting domains, named LiDs [87].

Later, the DamID approach was exploited by Perovanovic et al. to demonstrate that *LMNA* mutations affect the euchromatin-heterochromatin transitions during terminal differentiation of cells [88]. Hence, specific lamin A mutations were shown to cause promiscuous lamina-associated domains (LADs) and the inability to initiate and spread LADs.

Remarkably, ChIP was applied to murine dermal fibroblasts and showed that A-type lamins associate with both heterochromatin and euchromatin; the last interaction occurs outside of LADs, as demonstrated by Gesson and coworkers [89].

Some other applications of DamID in the field of lamins have been already reviewed in [90], whereas a detailed protocol for ChIP of lamin A/C and lamin B1 in human cells has been described in [91].

## 8. Conclusions

Recently, the interest in lamins has been remarkably fostered, especially after discovering that mutations in *LMNA* gene cause the onset of severe diseases like progeria and muscular dystrophies. Moreover, lamins have also started playing a key role in the cancer field. Because these disorders are life-threatening and effective therapeutic options are often not available, the scientific community is making great efforts to overcome the lack of knowledge about lamins. As seen from the present work, many different approaches have been pursued, ranging from conventional biology to the emerging field of biomechanics. Numerous techniques and procedures have been exploited thus far to elucidate the structure of lamins, their functions and involvement in the pathogenesis of certain diseases. Nonetheless, many unanswered questions will need to be answered before finding novel treatments that can actually cure the aforementioned pathologies. Research on lamins is paving the way towards new therapies, but awareness of what has already been done and pursued shall be the cornerstone of any project. This review contributes to the field by providing insight on useful techniques used to study lamins and, hopefully, suggests combined experimental tools to the scientists in the field.
